# Impact of Antibiotics Used for Acute Aspiration Bronchitis on the Prevention of Pneumonia

**DOI:** 10.3390/geriatrics9020026

**Published:** 2024-02-25

**Authors:** Akihiko Goto, Kosaku Komiya, Kenji Umeki, Kazufumi Hiramatsu, Jun-ichi Kadota

**Affiliations:** 1 Department of Respiratory Medicine, Tenshindo Hetsugi Hospital, 5956 Nihongi, Nakahetsugi, Oita 879-7761, Japan; 2 Department of Respiratory Medicine and Infectious Diseases, Oita University Faculty of Medicine, 1-1 Idaigaoka, Hasama-machi, Yufu 879-5593, Japan; 3 Department of Medical Safety Management, Oita University Faculty of Medicine, 1-1 Idaigaoka, Hasama-machi, Yufu 879-5593, Japan; 4 Nagasaki Harbor Medical Center, 6-39 Shinchi-machi, Nagasaki 850-8555, Japan

**Keywords:** aspiration bronchitis, aspiration pneumonia, antibiotics

## Abstract

Backgrounds: It remains unclear if antibiotics should be used for the treatment of acute aspiration bronchitis to prevent the development of pneumonia. This study aimed to assess the associations between the use of antibiotics and the development of pneumonia among patients with acute aspiration bronchitis. Methods: We retrospectively reviewed consecutive patients with acute aspiration bronchitis aged ≥75 years. Acute aspiration bronchitis was defined as a condition with aspiration risk, high fever (body temperature, ≥37.5 °C), respiratory symptoms, and the absence of evidence of pneumonia. Results: There was no significant difference in the incidence of pneumonia between patients treated with and without antibiotics for acute aspiration bronchitis (6/44, 14% vs. 31/143, 22%; *p* = 0.242). Lower estimated glomerular filtration rate (adjusted odds ratio, 0.956; 95% confidence interval, 0.920–0.993) was significantly associated with the development of pneumonia. Conclusions: Antibiotic administration should not be routinely recommended to prevent pneumonia following acute aspiration bronchitis, and patients with decreased renal function should be closely monitored. A randomized controlled trial is necessary to validate these results.

## 1. Introduction

Antimicrobial resistance (AMR) is a global human and animal health concern. The increasing incidence of AMR is predicted to cause 10 million mortalities annually by 2050 [1]. In 2015, the World Health Organization declared that AMR is one of the top 10 global public health threats that humanity is facing, and a global action plan has been adopted for AMR. The plan aims to address AMR and achieve the Sustainable Development Goals. Because of inadequate regulation and inappropriate use of antibiotics, which lead to AMR, this action plan is focused on the optimization of the use of antibiotics [2]. Notably, the rate of isolation of antibiotic-resistant bacteria and the extent of antibiotic use are positively correlated. For example, surveillance data from the European Antimicrobial Resistance Surveillance System indicate that the resistance of *Staphylococcus pneumoniae* to penicillin was correlated with the use of beta-lactam antibiotics and macrolides [3]. Another study from Germany indicated that the use of imipenem was a major factor associated with both carbapenem and beta-lactam resistance in endemic *Pseudomonas aeruginosa* [4]. Consequently, the indication for requirement of antibiotics should be carefully determined.

The aging population has increased globally because of an increase in life expectancy [5]. This has resulted in a gradual decrease in physical and mental capacity, and a growing risk of disease and death. Pneumonia is a major infectious disease affecting older people, and its prevalence among aging populations is increasing. Aspiration pneumonia is a common condition observed among older patients with community-acquired pneumonia (CAP) or hospital-acquired pneumonia [6], and is diagnosed when a patient at risk of aspiration develops pneumonia [7]. Risk factors for aspiration include impaired swallowing (esophageal diseases, chronic obstructive pulmonary disease, neurologic disease, and mechanical ventilation), impaired consciousness (neurologic disease, cardiac arrest, medication, general anesthesia, and alcohol consumption), increased chance of gastric contents reaching the lung (reflex and tube feeding), and impaired cough reflex (medication, alcohol, stroke, dementia, degenerative neurologic disease, and impaired consciousness) [8].

Conversely, acute aspiration bronchitis is diagnosed when a patient at risk of aspiration exhibits respiratory symptoms but does not show any evidence of pneumonia [9]. Acute bronchitis is generally characterized by a persistent cough and is diagnosed when no infiltration on chest radiographs or CT is observed. In most cases, the cough persists for a few weeks and then usually resolves spontaneously [10]. Viruses such as influenza A and B, rhinoviruses, coronaviruses, adenoviruses, respiratory syncytial viruses, human metapneumoviruses, and parainfluenza viruses account for approximately 90% of the causative pathogens. Additionally, *Bordetella pertussis*, *Mycoplasma pneumoniae*, and *Chlamydophila pneumoniae* account for approximately 10% of the causative pathogens [11]. There is no evidence that other bacterial infections directly cause acute bronchitis in adults without any underlying disease [10]. Patients who were given antibiotics for acute bronchitis in the absence of underlying disease or with mild underlying disease (e.g., hypertension, dyslipidemia, well-controlled diabetes) exhibited a shorter mean cough duration but had increased adverse effects associated with antibiotic treatments, such as diarrhea [12]. Moreover, there is insufficient evidence regarding the ability of chest X-ray to exclude pneumonia and tuberculosis or detect the causative bacteria for antimicrobial administration. For cases in which the cough intensity has passed its peak, follow-up is recommended as the patient is considered to have postinfectious cough, but antibiotics administration is not recommended. The absence of abnormalities in vital signs (heart rate ≥ 100 beats/min, respiratory rate ≥ 24 breaths/min, or oral temperature ≥ 38 °C) and chest examination (focal consolidation, e.g., rales, egophony, or fremitus) sufficiently reduces the risk of pneumonia, thereby disregarding the need for further diagnostic testing [13]. Alternatively, when a chest X-ray shows normal findings, physicians need to distinguish pertussis, mycoplasma infection, and *Chlamydophila* infection as acute bronchitis, for which antibiotics should be considered.

Most studies that examine the usefulness of antibiotics in acute bronchiolitis have excluded patients with underlying diseases. Since the underlying diseases include respiratory, cardiovascular, and renal diseases, and their severity differs, it becomes difficult to establish a standardized indication for antimicrobials. Acute bronchitis in patients with underlying pulmonary diseases, such as chronic obstructive pulmonary disease (COPD), bronchiectasis, and old pulmonary tuberculosis, is often associated with bacterial infection. In these cases, antibiotic treatment is recommended as it can increase clinical cure rates and reduce disease mortality [14,15]. In patients with COPD, purulent sputum is more likely to indicate a bacterial infection and the use of antibiotics is recommended [16]. *Haemophilus influenzae*, *S. pneumoniae*, *P. aeruginosa*, and *Staphylococcus aureus* are the most common causative bacteria in cases of COPD exacerbation [17]. For cases with suspected lower respiratory tract infection in the presence of an underlying disease, chest X-rays should be performed to exclude pneumonia.

As mentioned earlier, there is an increasing incidence of aspiration-associated diseases, including aspiration pneumonia, worldwide, since older people are susceptible to swallowing dysfunctions. Nevertheless, indications for administering antibiotics for acute aspiration bronchitis have not been established. AMR measures are required to be addressed multidirectionally, and it is important to determine the necessity of antibiotic administration in patients with acute aspiration bronchitis to prevent AMR and alleviate the risk of pneumonia. Therefore, we aimed to assess the impact of antibiotic treatment on the incidence of pneumonia among patients diagnosed with acute aspiration bronchitis and determine risk factors for pneumonia development following acute aspiration bronchitis.

## 2. Material and Methods

### 2.1. Patients and Study Design

This retrospective cohort study was conducted at Tenshindo Hetsugi Hospital, a community hospital with 188 beds in Oita, Oita Prefecture, Japan. We retrospectively reviewed consecutive patients with acute aspiration bronchitis who were aged ≥75 years. We targeted patients aged ≥75 years because they constitute a majority of patients who develop aspiration pneumonia in Japan. To ensure the objectivity of the diagnosis of acute aspiration bronchitis or pneumonia, we focused on hospitalized patients who had completed the treatment for CAP between January 2018 and December 2021, were at risk for aspiration, remained hospitalized for rehabilitation, eventually developed a high fever (≥37.5 °C) with respiratory symptoms, and showed no evidence of pneumonia. Patients with fever from causes other than pneumonia were excluded. Patients diagnosed with pneumonia a day after experiencing fever were also excluded because they may have already developed pneumonia at the time when high fever was confirmed. The study protocol was in accordance with the ethical guidelines of the 1975 Declaration of Helsinki and was approved by the institutional ethics committee of Oita University Faculty of Medicine (approval number 2463; approval date 26 January 2023). The need for obtaining informed consent was waived by the committee because of the retrospective design of the study. This work was supported by Daiwa Securities Foundation Research Foundation 2022. The funding source was not involved in the conducting of the study, data collection, management, data analysis, interpretation, or manuscript preparation, review, or approval. The corresponding author has full access to all the data pertaining to this study and holds final responsibility for the decision to submit it for publication. Some patients included in this study were also included in previous studies [18,19,20,21].

### 2.2. Definitions and Outcomes

CAP was defined based on the criteria developed by the Infectious Diseases Society of America/American Thoracic Society guidelines [22]. These criteria are based on clinical signs and symptoms of patients, such as fever and cough, and the detection of infiltrates using chest radiography or computed tomography. Acute aspiration bronchitis was defined as a febriferous condition with aspiration risk and respiratory symptoms, including cough and sputum, but without observation of infiltration on chest radiography or patients in whom the physician did not suspect pneumonia. Aspiration risks included impaired consciousness, cerebrovascular disease, or decreased level of daily physical activity (ADL) assessed as a Barthel Index value of <60 [8]. A previous study identified a Barthel Index value of <60 as an independent predictor of aspiration risk [23]. Respiratory failure was defined as an oxygen saturation of <90% without supplemental oxygen. The estimated glomerular filtration rate (eGFR) was calculated using serum creatinine levels and age [24]. This is the calculation formula used for Japanese patients aged 18 and over. The modification of diet in renal disease study group developed a GFR estimation formula based on the following six parameters: serum creatinine level, serum urea level, serum albumin level, age, sex, and race (Black or not) [25]. As muscle volume and other factors differ between Japanese and Westerners, it was necessary to create a formula that is optimized for Japanese population. Using the above formula, it is possible to simply and accurately assess renal function in Japanese patients. The primary outcome was the proportion of patients who developed pneumonia after treatment with or without antibiotics within 7 days following the diagnosis of acute aspiration bronchitis. Pneumonia following acute aspiration bronchitis was also diagnosed based on the criteria proposed in the guidelines [22]. We compared the proportion of pneumonia development with baseline characteristics in patients with acute aspiration bronchitis who were treated with and without antibiotics. In addition, the factors associated with the development of pneumonia were assessed via univariate and multivariate analyses.

### 2.3. Data Collection

Patients’ data including sex, age, body mass index (BMI), underlying diseases (COPD, cardiac disease, cerebrovascular disease, and diabetes mellitus), systolic blood pressure, laboratory data, presence of impaired consciousness and respiratory failure, 30-day mortality after diagnosis of acute aspiration bronchitis, and chest radiographic findings were obtained from clinical records. Systolic blood pressure and the presence of respiratory failure on the day of fever were documented. Laboratory data were obtained within 1 day after developing acute aspiration bronchitis. We evaluated patients’ ADL level using the Barthel Index. The Barthel Index was introduced in 1965 and originally established as a 0–20 scale [26]. It was modified by Granger et al. in 1979 to include 0–10 points for each item (i.e., a total possible score of 0–100) [27]. Information gathered about 10 basic activities of daily living was obtained through the revised Barthel Index: bowels, bladder, grooming, toilet use, feeding, transfers, walking, dressing, climbing stairs, and bathing. For the Barthel Index, a score of 100 is regarded as fully independent, 60 as partially independent, and 0 as requiring full assistance. The Barthel Index is used worldwide, because of the simplicity of ADL assessment and the less time required for the decision-making process. Information regarding antimicrobial use for acute aspiration bronchitis and not for pneumonia along with pneumonia development within 7 days after the diagnosis of acute aspiration bronchitis was documented. The routine collection of patients’ data and information regarding their examination performance is recommended when a patient diagnosed with CAP is admitted to our hospital.

### 2.4. Statistical Analyses

Statistical analyses were performed using the Statistical Package for the Social Sciences software version 28 (IBM Japan, Tokyo, Japan). Continuous variables were expressed as medians (interquartile range) and categorical variables were presented as numbers and percentages. The confidence intervals (CIs) in the two-sided analyses were 95%. Statistical significance was defined as a *p*-value < 0.05 for all analyses. Continuous variables were compared using the Mann–Whitney test, whereas categorical variables were compared via the chi-squared test or Fisher’s exact test between patients who were treated with and without antibiotics. Logistic regression analysis was performed to assess factors associated with pneumonia development following acute aspiration bronchitis, and crude odds ratio (OR) and *p*-value were calculated. Variables with a *p*-value of ≤0.1 in the univariate analysis were used for multivariate analysis. This analysis was conducted to assess the impact of each predictor variable on the results and to determine the statistical significance of this impact.

## 3. Results

**Figure 1:**
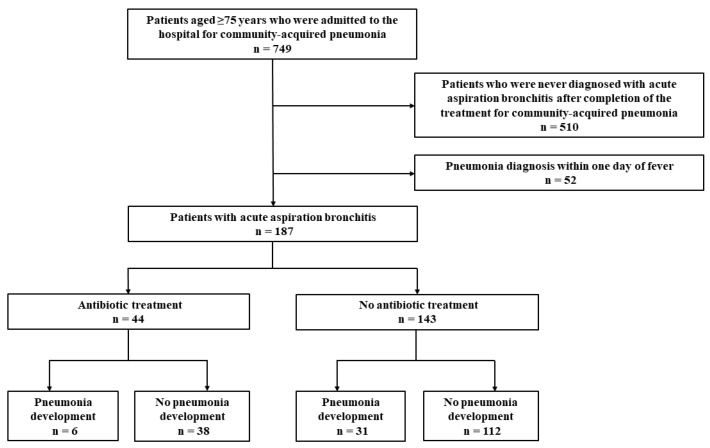
Patient flow diagram.

We initially screened 749 patients aged ≥75 years who were admitted to the hospital for CAP during the study period. Furthermore, 510 patients who were never diagnosed with acute aspiration bronchitis after completion of the treatment for CAP were excluded, including 21 patients with fever due to causes other than aspiration bronchitis. Of the remaining 239 patients, we excluded 52 patients who were diagnosed with pneumonia within 1 day of developing fever. Finally, 187 patients were included in the study (Figure 1).

Of the 187 patients who were diagnosed with acute aspiration bronchitis, 44 received antibiotics; subsequently, 6 (14%) developed pneumonia and 38 (86%) did not. Conversely, 143 patients did not receive antibiotics for aspiration bronchitis; 31 (22%) eventually developed pneumonia and 112 (78%) did not. There was no significant difference in the incidence of pneumonia between patients treated with and without antibiotics (*p* = 0.242). The antibiotics used among patients who were treated with antibiotics included sulbactam/ampicillin (n = 10, 23%), levofloxacin (n = 8, 18%), tazobactam/piperacillin (n = 7, 16%), ceftriaxone (n = 6, 14%), cefmetazole (n = 2, 5%), amoxicillin hydrate/potassium clavulanate (n = 2, 5%), cefditoren pivoxil (n = 2, 5%), cefcapene pivoxil (n = 2, 5%), cefazolin (n = 1, 2%), sulbactam/cefoperazone (n = 1, 2%), azithromycin (n = 1, 2%), vancomycin (n = 1, 2%), garenoxacin (n = 1, 2%), and sulfamethoxazole/trimethoprim (n = 1, 2%). One patient was treated with a combination of antibiotics (overlap permitted). The median duration of antimicrobial treatment was 5 (interquartile range, 4–6) days. Blood examinations were more frequently performed within 1 day after the diagnosis of aspiration bronchitis in patients treated with antibiotics than in those treated without antibiotics (25/44, 57% in the antibiotic treatment group vs. 41/143, 29% in the nonantibiotic treatment group). The antibiotic treatment group comprised significantly younger patients (median, 84 vs. 89; *p* = 0.003), a higher number of males (70% vs. 45%; *p* = 0.004), patients with higher BMI (19.3 vs. 17.7; *p* = 0.028), and patients with a higher white blood cell (WBC) count (9070 vs. 6240; *p* = 0.003) and C-reactive protein (CRP) level (3.4 vs. 1.2; *p* = 0.009) than the nonantibiotic treatment group (Table 1). There was no significant difference in the 30-day mortality following the diagnosis of acute aspiration bronchitis (5% vs. 10%; *p* = 1.000).

Lower eGFR (OR, 0.957; 95% CI, 0.922–0.993; *p* = 0.019) was significantly related, whereas antibiotics use for aspiration bronchitis was not related (OR, 0.570; 95% CI, 0.221–1.473; *p* = 0.246), to pneumonia development following diagnosis of aspiration bronchitis (Table 2). Blood examinations within 1 day after diagnosis of acute aspiration bronchitis were more frequently performed in patients who did not develop pneumonia than in those who developed pneumonia (33% vs. 13%). Patients who developed pneumonia tended to have lower systolic blood pressure and higher CRP levels, but no statistically significant difference was observed. There was no significant difference in the 30-day mortality after the diagnosis of acute aspiration bronchitis between patients who developed pneumonia and those who did not (11% vs. 11%; *p* = 1.000). In multivariate analysis, after adjustment for systolic blood pressure and CRP, lower eGFR (OR, 0.956; 95% CI, 0.920–0.993; *p* = 0.020) was significantly associated with pneumonia development (Table 3).

## 4. Discussion

This study demonstrated that there is no significant difference in the incidence of development of pneumonia between patients with acute aspiration bronchitis who were treated with and without antibiotics. Multivariate analysis showed that lower eGFR was significantly associated with pneumonia development.

Owing to the retrospective and observational nature of this study, patients’ backgrounds and clinical conditions at the time of acute aspiration bronchitis diagnosis differed between patients treated with and without antibiotics. Patients who received antibiotics were predominantly male and younger and had higher BMI, WBC count, and CRP levels than those who did not receive antibiotics. These results indicate that physicians were likely to prescribe antibiotics to younger males with high inflammatory status. Older patients may frequently experience repeated aspiration-associated events, and the attending physicians may hesitate to prescribe antibiotics. Being male and having a high inflammatory status are known risk factors for severe progression of pneumonia, which may be considered in the decision-making for antibiotics indication [28]. Nevertheless, no significant difference was observed in the incidence of pneumonia and 30-day mortality between patients who were treated with and without antibiotics. These findings indicate that antibiotics administration for the treatment of acute aspiration bronchitis is not associated with the prevention of pneumonia, at least for older patients with low inflammatory status.

Low blood pressure and eGFR might reflect a hypovolemic status or the presence of bacteremia [29]. Blood pressure and renal function are included as items in the assessment of severity of CAP based on the CURB-65 score [30]. The usefulness of the scoring system was evaluated among older patients who were hospitalized for CAP [31]. The results indicated that the CURB score predicted 30-day mortality with the same accuracy as Pneumonia Severity Index class did among older adults hospitalized for CAP. Therefore, blood pressure and renal function may be useful markers for stratifying disease severity, even for elderly patients with pneumonia. As low eGFR was associated with pneumonia development in the present study, it may predict the progression from acute aspiration bronchitis to pneumonia.

In the current study, CRP levels in patients who developed pneumonia tended to be higher than those in patients who did not. Previously, we have demonstrated that high CRP levels were significantly associated with in-hospital mortality among patients with aspiration pneumonia [32]. CRP levels may predict disease progression as well as the development of aspiration pneumonia. A systematic review evaluating the accuracy of procalcitonin (PCT) and CRP levels for the diagnosis of bacterial infection revealed that PCT level was more sensitive (88%, 95% CI 80–93% vs. 75%, 95% CI 62–84%) and more specific (81%, 95% CI 67–90% vs. 67%, 95% CI 56–77%) compared with CRP level in differentiating bacterial from non-infectious causes of inflammation [33]. The accuracy of diagnosis using PCT level was higher compared with that using CRP levels among patients hospitalized for suspected bacterial infections; however, cost-effective analyses are necessary for encouraging the use of PCT instead of CRP measurements. Furthermore, there is a time lag between the onset of symptoms and the increase in the value of these biomarkers. The timing of the measurements appears to vary according to the area or institutions, and physicians are required to consider them when assessing the possibility of bacterial infections based on the values. Although the current observational study cannot clearly establish the indications for the antibiotics use among patients with acute aspiration bronchitis, patients with decreased renal function and an elevated inflammatory status should be closely monitored, as these are high-risk factors for the subsequent development of pneumonia.

The strength of this study is that this is the first report where the impact of antibiotics use for acute aspiration bronchitis on the prevention of pneumonia was assessed. However, ventilator-related bronchitis may be conceptually similar to acute aspiration bronchitis [34], which has an incidence similar to that of ventilator-associated pneumonia, with a high prevalence of isolated multidrug-resistant pathogens. This results in an increase in the time of mechanical ventilation and hospitalization, but without an impact on mortality. The performance of quantitative cultures may provide a better diagnostic definition of tracheobronchitis associated with mechanical ventilation, possibly avoiding the overdiagnosis of this condition. Clinical practice guidelines developed by the Infectious Diseases Society of America and American Thoracic Society discourage antibiotic administration for ventilator-related bronchitis despite the lack of solid evidence [35]. The guidelines provided evidence that the treatment for ventilator-associated tracheobronchitis improves clinical outcomes, which is required to determine if the treatment for ventilator-associated tracheobronchitis is warranted. A randomized controlled trial that assigned 58 patients diagnosed with ventilator-associated tracheobronchitis to receive either intravenous antibiotics or no antibiotics for 8 days demonstrated that antimicrobial treatment is associated with a higher number of days that are free of mechanical ventilation with lower rates of VAP and ICU mortality; however, antibiotic treatment has no significant impact on the total duration of mechanical ventilation [36]. Additionally, several observational studies compared adult mechanically ventilated patients with ventilator-associated tracheobronchitis receiving intravenous antibiotics to those who did not receive antibiotics [36,37,38,39]. Antibiotic therapy was associated with a shorter duration of mechanical ventilation, but no significant differences were observed in mortality or duration of ICU stay. Based on these findings the guidelines suggest not to administer antibiotic therapy (weak recommendation, low-quality evidence) [35]. We observed no significant association between antibiotic treatment for acute aspiration bronchitis and subsequent pneumonia development in the current analyses; however, other outcomes were not evaluated. Therefore, no recommendation of antibiotic treatment for acute aspiration bronchitis seems to be reasonable, which is consistent with the description of ventilator-associated tracheobronchitis in the guidelines.

However, this study has some limitations to discuss. First, as mentioned earlier, the decision to use antibiotics may vary among attending physicians, leading to selection bias in this retrospective observational study. We compared the baseline characteristics between patients who were treated with and without antibiotics for acute aspiration bronchitis; however, other variables that were not documented (e.g., patient’s desire to receive antibiotics) may differ and affect the physician’s intention to prescribe antibiotics. Second, we could obtain the results of blood examination performed within 1 day of acute aspiration bronchitis diagnosis for only one-third of the patients included in this study. This study did not conduct missing value analysis because the number of patients who did not undergo blood tests was lower than that of patients who did, making it challenging to estimate missing values [40]. Interestingly, patients who did not receive antibiotics and did not develop pneumonia were more likely to have undergone blood examination. This suggests that low CRP levels can help in ruling out the risk for developing pneumonia. In other words, patients at risk for pneumonia might require more frequent blood examination. However, as mentioned earlier, there is a time lag between the onset of symptoms and increased CRP levels. Further studies are required to assess the optimal timing of CRP measurements for predicting pneumonia development in patients with acute aspiration bronchitis. Third, pneumonia was diagnosed based on chest X-ray results and symptoms observed in patients in this study. In fact, chest X-ray was performed to diagnose aspiration pneumonia for all patients, but it was not used to rule out pneumonia during the diagnosis of aspiration bronchitis in all cases. If the attending physician did not suspect pneumonia, chest X-ray was not performed, which may have led to assessment bias. Additionally, chest X-ray-negative pneumonia can account for pneumonia occurring in patients with bed-ridden status [41]. Older individuals with risk factors for aspiration generally have poor physical activity levels. Thus, some people may have been underdiagnosed for pneumonia. Fourth, it is impossible to distinguish aspiration pneumonitis from aspiration pneumonia. Technically, aspiration pneumonitis need not be treated with antibiotics, which may also lead to assessment bias. Finally, this study was conducted in a single center with a limited number of patients, which limits the generalizability of the obtained results. However, to ensure the objectivity of the diagnosis of acute aspiration bronchitis or pneumonia, we focused on patients who were hospitalized and had completed treatment for CAP at our institution. This environment will be suitable to carefully observe the progression from acute bronchitis to pneumonia.

In conclusion, this study showed no significant difference in the incidence of pneumonia between patients with acute aspiration bronchitis who were treated with and without antibiotics. Antibiotics administration is not routinely recommended for the treatment of acute aspiration bronchitis. Considering the result that low eGFR is associated with pneumonia development, patients who develop aspiration bronchitis with low renal function should be carefully monitored. However, the results of this study may have been biased due to the retrospective nature of this study. A randomized controlled trial is warranted to validate these results and identify the characteristics of patients who should be treated with antibiotics for acute aspiration bronchitis to prevent pneumonia.

## Figures and Tables

**Table 1:** Baseline characteristics of patients with acute aspiration bronchitis who were treated with or without antibiotics.

	Antibiotic Treatment (n = 44)	No Antibiotic Treatment (n = 143)	*p*-Value
Female (number with percentage)	13 (30)	78 (55)	0.004
Age (years, median with interquartile range)	84 (82–90)	89 (84–93)	0.003
BMI (kg/m^2^, median with interquartile range)	19.3 (16.4–21.1)	17.7 (15.4–20.6)	0.028
Barthel Index (median with interquartile range)	0 (0–10)	0 (0–10)	0.664
Barthel Index < 60 (number with percentage)	41 (93)	141 (99)	0.086
Systolic blood pressure (mmHg, median with interquartile range)	117 (99–128)	116 (99–129)	0.878
Impaired consciousness (number with percentage)	24 (55)	94 (66)	0.179
Respiratory failure (number with percentage)	14 (32)	31 (22)	0.169
WBC count (/μL, median with interquartile range)	9070 (6450–10,380)	6240 (5320–7460)	0.003
Hemoglobin (g/dL, median with interquartile range)	11.1 (9.6–11.9)	11.1 (9.7–11.9)	0.968
Albumin (g/dL, median with interquartile range)	2.6 (2.2–3.0)	2.7 (2.4–3.1)	0.285
ALT (IU/L, median with interquartile range)	12 (10–16)	14 (10–21)	0.194
eGFR (mL/min/1.73 m^2^, median with interquartile range)	72.4 (45.2–96.9)	67.4 (49.8–95.9)	0.995
CRP (mg/dL, median with interquartile range)	3.4 (2.0–7.4)	1.2 (0.5–4.2)	0.009
COPD (number with percentage)	5 (11)	18 (13)	0.829
Cardiac disease (number with percentage)	15 (34)	40 (28)	0.436
Cerebrovascular disease (number with percentage)	17 (39)	47 (33)	0.481
Diabetes mellitus (number with percentage)	10 (23)	28 (20)	0.650
Pneumonia development (number with percentage)	6 (14)	31 (22)	0.242
30-day mortality after the diagnosis of acute aspiration bronchitis (number with percentage)	5 (11)	15 (10)	1.000

ALT, alanine aminotransferase; BMI, body mass index; COPD, chronic obstructive pulmonary disease; CRP, C-reactive protein; eGFR, estimate glomerular filtration rate; WBC, white blood cell.

**Table 2:** Univariate analysis of factors associated with the development of pneumonia.

	Patients Who Developed Pneumonia (n = 37)	Patients Who Did Not Develop Pneumonia (n = 150)	Crude Odds Ratio	*p*-Value
Female (number with percentage)	16 (43)	75 (50)	0.762 (0.369–1.573)	0.462
Age (years, median with interquartile range)	87 (84–92)	88 (83–92)	0.989 (0.928–1.054)	0.732
BMI (kg/m^2^, median with interquartile range)	18.0 (15.0–20.2)	18.2 (15.8–20.8)	0.958 (0.863–1.062)	0.414
Barthel Index (median with interquartile range)	0 (0–15)	0 (0–10)	0.999 (0.978–1.020)	0.914
Barthel Index < 60 (number with percentage)	37 (100)	145 (97)	NA	0.999
Systolic blood pressure (mmHg, median with interquartile range)	109 (97–121)	118 (101–131)	0.984 (0.965–1.003)	0.092
Impaired consciousness (number with percentage)	22 (59)	96 (64)	0.825 (0.395–1.722)	0.609
Respiratory failure (number with percentage)	11 (30)	34 (23)	1.443 (0.647–3.219)	0.370
WBC count (/μL, median with interquartile range)	7215 (5870–9715)	6490 (5378–9070)	1.000 (1.000–1.000)	0.145
Hemoglobin (g/dL, median with interquartile range)	10.6 (9.1–11.0)	11.2 (9.7–11.9)	0.900 (0.575–1.408)	0.644
Albumin (g/dL, median with interquartile range)	2.9 (2.6–3.2)	2.7 (2.4–3.0)	2.306 (0.284–18.703)	0.434
ALT (IU/L, median with interquartile range)	12 (11–14)	14 (10–20)	0.980 (0.880–1.091)	0.706
eGFR (mL/min/1.73 m^2^, median with interquartile range)	27.3 (15.1–56.4)	71.2 (50.0–98.4)	0.957 (0.922–0.993)	0.019
CRP (mg/dL, median with interquartile range)	8.1 (3.6–9.6)	2.0 (0.9–5.4)	1.178 (0.981–1.414)	0.079
COPD (number with percentage)	4 (11)	19 (13)	0.836 (0.266–2.623)	0.758
Cardiac diseases (number with percentage)	9 (24)	46 (31)	0.727 (0.318–1.662)	0.449
Cerebrovascular disease (number with percentage)	10 (27)	54 (36)	0.658 (0.296–1.463)	0.305
Diabetes mellitus (number with percentage)	7 (19)	31 (21)	0.896 (0.360–2.231)	0.813
Use of antibiotics (number with percentage)	6 (16)	38 (25)	0.570 (0.221–1.473)	0.246

ALT, alanine aminotransferase; BMI, body mass index; COPD, chronic obstructive pulmonary disease; CRP, C-reactive protein; eGFR, estimate glomerular filtration rate; NA, not available; WBC, white blood cell.

**Table 3:** Multivariate analysis of factors associated with the development of pneumonia.

	Adjusted Odds Ratio	*p*-Value
Systolic blood pressure (mmHg)	0.990 (0.946–1.038)	0.687
eGFR (mL/min/1.73 m^2^)	0.956 (0.920–0.993)	0.020
CRP (mg/dL)	1.190 (0.976–1.452)	0.086

CRP, C-reactive protein; eGFR, estimate glomerular filtration rate.

## Data Availability

The data that support the findings of this study are available from the corresponding author, upon reasonable request.

## Abbreviations

AMR	Antimicrobial resistance
CAP	Community-acquired pneumonia
CI	Confidence intervals
CRP	C-reactive protein
eGFR	Estimated glomerular filtration rate
WBC	White blood cell
WHO	World Health Organization
